# Sex Differences in Outcome After Thrombectomy for Acute Ischemic Stroke are Explained by Confounding Factors

**DOI:** 10.1007/s00062-020-00983-2

**Published:** 2020-12-21

**Authors:** Milani Deb-Chatterji, Eckhard Schlemm, Fabian Flottmann, Lukas Meyer, Anna Alegiani, Caspar Brekenfeld, Jens Fiehler, Christian Gerloff, Götz Thomalla, GSR-ET Investigators, C. Gerloff, J. Fiehler, G. Thomalla, A. Alegiani, Boeckh-Behrens, Silke Wunderlich, Ulrike Ernemann, Sven Poli, Eberhard Siebert, Christian H. Nolte, Sarah Zweynert, Georg Bohner, Alexander Ludolph, Karl-Heinz Henn, Waltraud Pfeilschifter, Fee Keil, Joachim Röther, Bernd Eckert, Jörg Berrouschot, Albrecht Bormann, László Solymosi, Gabor Petzold, Christoffer Kraemer, Martin Dichgans, Steffen Tiedt, Lars Kellert, Franziska Dorn, Martina Petersen, Florian Stögbauer, Michael Braun, Gerhard F. Hamann, Klaus Gröschel, Timo Uphaus

**Affiliations:** 1 Department of Neurology, University Medical Center Hamburg-Eppendorf, Hamburg, Germany; 2 Department of Interventional Neuroradiology and Diagnostics, University Medical Center Hamburg-Eppendorf, Hamburg, Germany

**Keywords:** Ischemia, Gender difference, Outcome predictors, Intervention, Angiography

## Abstract

**Purpose:**

The aim of this study was to analyze sex differences in outcome after thrombectomy for acute ischemic stroke in clinical practice in a large prospective multicenter registry.

**Methods:**

Data of consecutive stroke patients treated with thrombectomy (June 2015–April 2018) derived from an industry-independent registry (German Stroke Registry–Endovascular Treatment) were prospectively analyzed. Multivariable binary logistic regression analyses were applied to determine whether sex is a predictor of functional independence outcome (defined as a modified Rankin scale [mRS] 0–2) 90 days after stroke.

**Results:**

In total, 2316 patients were included in the analysis, 1170 (50.5%) were female and 1146 (49.5%) were male. Women were older (median age 78 vs. 72 years; *p* < 0.001) and more frequently had a prestroke functional impairment defined by mRS >1 (24.8% vs. 14.1%; *p* < 0.001). In unadjusted analyses, independent outcome at 90 days was less frequent in women (33.2%) than men (40.6%; *p* < 0.001). Likewise, mortality was higher in women than in men (30.7% vs. 26.4%; *p* = 0.024). In adjusted regression analyses, however, sex was not associated with outcome. Lower age, a lower baseline National Institutes of Health Stroke Scale score, a higher Alberta Stroke Program Early CT score, prestroke functional independence, successful reperfusion, and concomitant intravenous thrombolysis therapy predicted independent outcome.

**Conclusion:**

Women showed a worse functional outcome after thrombectomy for acute ischemic stroke in clinical practice; however, after adjustment for crucial confounders sex was not a predictor of outcome. The difference in outcome thus appears to result from differences in confounding factors such as age and prestroke functional status.

**Supplementary Information:**

The online version of this article (10.1007/s00062-020-00983-2) contains supplementary material, which is available to authorized users.

## Introduction

Multiple randomized controlled trials (RCTs) have demonstrated the efficacy of endovascular treatment (EVT) among patients with acute ischemic stroke caused by large vessel occlusions. Several studies have reported worse outcome for females after acute ischemic stroke compared to males [1–5]; however, conflicting results were reported on sex differences in outcome after stroke thrombectomy.

Post hoc analyses of RCTs on stroke thrombectomy within the HERMES collaboration suggested that sex does not influence functional outcome after EVT and demonstrated a similar proportion of both women and men, achieving functional independence 90 days after intervention [6]. Similar results were obtained in a pooled analysis of patients from different trial cohorts, which did not find a gender difference in outcome at 90 days, although females were older and had higher rates of atrial fibrillation [7]. Conversely, an analysis of the MR CLEAN trial reported that women were less likely to benefit from EVT with a lower proportion of independence before stroke and higher rates of serious adverse events and mortality in women than in men [8]. Thus, it is still largely unknown whether outcome after stroke thrombectomy differs between women and men. Of note, comparable studies in nonclinical trial populations are scarce. Identifying sex differences in outcome after EVT in a real-world setting may lead to targeted approaches for patient selection or sex-specific rehabilitation therapies.

The objective of our study was to determine whether sex is associated with outcome of stroke thrombectomy in clinical practice.

## Methods

### Data Collection

The patients analyzed in this study were derived from the German Stroke Registry—Endovascular Treatment (GSR-ET; ClinicalTrials.gov, identifier: NCT03356392). The GSR-ET is an ongoing prospective, multicenter registry of 25 participating sites in Germany including acute ischemic stroke patients with proximal large vessel occlusion of the anterior and posterior circulation treated by EVT. The study design and major findings had been reported previously [9, 10]. For this analysis, we included patients enrolled in the registry between June 2015 and April 2018.

The decision for EVT was made by an interdisciplinary team including vascular neurologists and interventional neuroradiologists on a case by case basis. Patients were older than 18 years.

Patients’ age, the time elapsed from symptom onset to imaging, baseline National Institutes of Health Stroke Scale (NIHSS) score, occlusion site, the Alberta Stroke Program Early CT Score (ASPECTS), the prestroke disability assessed by the modified Rankin scale (mRS), the results of multiparametric imaging with computed tomography perfusion or magnetic resonance imaging to identify potentially salvageable brain tissue were all considered for treatment decision. Intravenous thrombolysis therapy (IVT) was applied prior to intervention as appropriate according to national and international guidelines [11, 12]. Imaging data were analyzed and reported by the local centers.

The mRS score of the patients was collected 3 months after the stroke and provided by each study site. The mRS was assessed by a well-trained investigator, who was blinded to patient variables including the results of EVT.

Patients with missing data on follow-up assessments at 90 days were excluded from this subgroup analysis.

### Statistical Analysis

Standard descriptive statistics were reported as median and interquartile range or continuous variables and numbers and percentages for categorical variables. For between-group comparisons of categorical variables, χ^2^-tests or Fisher’s exact tests were used, as appropriate. Mann-Whitney *U*-tests were employed for continuous variables.

Multivariable binary logistic regression analyses were applied to identify predictors of an independent outcome (defined as mRS 0–2) and of death (mRS 6) 90 days after stroke, and whether sex was associated with outcome. Moreover, ordinal regression analysis was performed to identify predictors of a worse outcome assessed by higher mRS scores at 90 days.

Besides sex, particular variables, which were known predictors of outcome after stroke thrombectomy, were included: age, NIHSS score on admission, ASPECTS as continuous independent variables, and concomitant IVT, successful reperfusion (defined by modified treatment in cerebral ischemia score [mTICI] 2b/3) and prestroke functional impairment assessed by a mRS >1 as dichotomous independent variables. The time from symptom onset to recanalization was reported in less than 75% of all subjects, so that the variable was excluded from the primary analysis.

Since the variable ASPECTS had more than 10% missing values, the analyses were run in two settings that differed with respect to the included variables contained in the initial model and to the total amount of patients in which complete information on the different variables was available:

- Model 1: containing all independent variables except ASPECTS (*n* = 2151)
- Model 2: containing all independent variables including ASPECTS (*n* = 1667)

The resulting odds ratios with 95% confidence intervals and *p* values are presented. Analysis was exploratory and *p* values <0.05 were considered statistically significant without correction for multiple testing. All tests were two-sided. Statistical analysis was performed using SPSS (Version 25.0; IBM, Armonk, NY, USA).

## Results

### Patient Data

In total, 2637 patients treated by EVT were consecutively enrolled in the registry during the study period. With respect to the primary outcome, data on follow-up assessment at 90 days were missing in 321 subjects, leaving 2316 patients for this analysis. The baseline data of the patients with missing outcome values did not differ from those with available 90-day follow-up information (Supplemental Table e1), indicating that a significant bias of patients lost to follow-up was not to be expected.

In the cohort of 2316 patients, 1170 patients (50.5%) were female and 1146 (49.5%) were male. Results of group comparisons by sex are displayed in Table 1.

**Table 1:** Patient characteristics by sex

	Females (*n* = 1170, 50.5%)	Males (*n* = 1146, 49.5%)	*P* value
*Age in years, median (IQR)*	78 (69–84)	72 (61–79)	*<0.001*
*Baseline NIHSS score, median (IQR)*	15 (10–19) (*n* = 1155)	15 (9–19) (*n* = 1133)	0.115
*Prestroke mRS >1, n (%)*	281/1131 (24.8)	155/1098 (14.1)	*<0.001*
*Living status before admission*			*<0.001*
Home, *n* (%)	924/1111 (83.2)	1117/1084 (93.8)	
Nursing at home, *n* (%)	64/1111 (5.8)	28/1084 (2.6)	
Nursing home, *n* (%)	123/1111 (11.1)	39/1084 (3.6)	
*Pre-existing comorbidities*			
Arterial hypertension, *n* (%)	916/1161 (78.9)	816/1135 (71.9)	*<0.001*
Hypercholesterolemia, *n* (%)	370/1160 (31.9)	404/1131 (35.7)	0.053
Diabetes mellitus, *n* (%)	234/1164 (20.1)	255/1135 (22.5)	0.166
Atrial fibrillation, *n* (%)	540/1162 (46.5)	393/1131 (34.7)	*<0.001*
*Drip ‘n ship, n (%)*	542/1170 (46.3)	561/1146 (49.0)	0.205
*Anterior circulation infarction, n (%)*	1029/1134 (90.7)	965/1122 (86.0)	*<0.001*
*ASPECTS, median (IQR)*	9 (7–10) (*n* = 901)	9 (7–10) (*n* = 863)	0.897
*IVT, n (%)*	649/1154 (56.2)	637/1141 (55.8)	0.843
*Symptom onset to groin puncture, median (IQR)*	195 (149.25–270) (*n* = 644)	197 (140.5–274.5) (*n* = 705)	0.616
*Symptom onset to recanalization, median (IQR)*	240 (190–318) (*n* = 525)	250 (183–322.5) (*n* = 597)	0.971
*TICI 2b/3, n (%)*	942/1140 (82.6)	934/1128 (82.8)	0.915
*Adverse events during treatment, n (%)* *(e.g. perforation, dissection)*	162/1157 (14.0)	139/1129 (12.3)	0.232
*NIHSS score 24h after treatment, median (IQR)*	11(4–20) (*n* = 1027)	10 (4–20) (*n* = 992)	0.093
*mRS score 24h after treatment, median (IQR)*	5 (3–5) (*n* = 1009)	5 (3–5) (*n* = 994)	0.076
*Adverse events 24h after treatment, n (%)* *(e.g. groin hematoma, malignant infarction)*	304/1146 (26.5)	294/1128 (26.1)	0.802
*Any ICH within 24* *h after treatment, n (%)*	162/1170 (13.8)	139/1146 (12.1)	0.219
*NIHSS score at discharge, median (IQR)*	5 (2–13) (*n* = 907)	5 (2–12) (*n* = 922)	0.480
*mRS score at discharge, median (IQR)*	4 (2–5) (*n* = 1140)	4 (2–5) (*n* = 1120)	0.065
*Adverse events until discharge, n (%)* *(e.g. infections, recurrent stroke)*	392/1105 (35.5)	384/1082 (35.5)	0.994
*Stroke etiology*			*<0.001*
Atrial fibrillation, *n* (%)	653/1152 (56.7)	487/1129 (43.1)	
Atherosclerosis, *n* (%)	236/1152 (20.5)	338/1129 (29.9)	
Other, *n* (%)	52/1152 (4.5)	59/1129 (5.2)	
Undetermined, *n* (%)	195/1152 (16.9)	206/1129 (18.2)	
Dissection, *n* (%)	16/1152 (1.4)	16/1129 (1.4)	
*Destination after discharge*			*<0.001*
Home, *n* (%)	214/925 (23.1)	252/927 (27.2)	
Neurorehabilitation, *n* (%)	507/925 (54.8)	515/927 (55.6)	
Hospital, *n* (%)	163/925 (17.6)	147/927 (15.9)	
Nursing home, *n* (%)	41/925 (4.4)	13/927 (1.4)	
*mRS at 90 days, median (IQR)*	4 (2–6)	3 (1–6)	*0.001*
*mRS 0–2* *at 90 days, n (%)*	389/1170 (33.2)	465/1146 (40.6)	*<0.001*
*mRS 5/6* *at 90 days, n (%)*	477/1170 (40.8)	417/1146 (36.4)	*0.030*
*mRS 6* *at 90 days, n (%)*	359/1170 (30.7)	303/1146 (26.4)	*0.024*

Significant values in italics

*IQR* interquartile range, *NIHSS* National Institutes of Health Stroke Scale, *mRS* modified Rankin scale, *IVT* intravenous thrombolysis,* ASPECTS* Alberta Stroke Program Early CT Score, *mTICI* modified thrombolysis in cerebral infarction score, *ICH* intracerebral hemorrhage

Women were older than men (median age 78 years vs. 72 years, *p* < 0.001), and were more likely to be diagnosed with the pre-existing comorbidities arterial hypertension (78.9% vs. 71.9%, *p* < 0.001) and atrial fibrillation (46.5% vs. 34.7%, *p* < 0.001). In addition, a higher proportion of women had a prestroke mRS >1 (24.8% vs. 14.1%, *p* < 0.001). Consistent with these findings, the living status before admission differed between both subgroups of patients (*p* < 0.001). A higher proportion of females received nursing care, either at home (5.8% vs. 2.6%) or at a nursing home (11.1% vs. 3.6%), instead of living at home (83.2% vs. 93.8%). Moreover, women suffered more frequently from vessel occlusions of the anterior circulation (90.7% vs. 86.0%, *p* < 0.001). Other baseline characteristics were comparable between women and men.

**Fig. 1:**
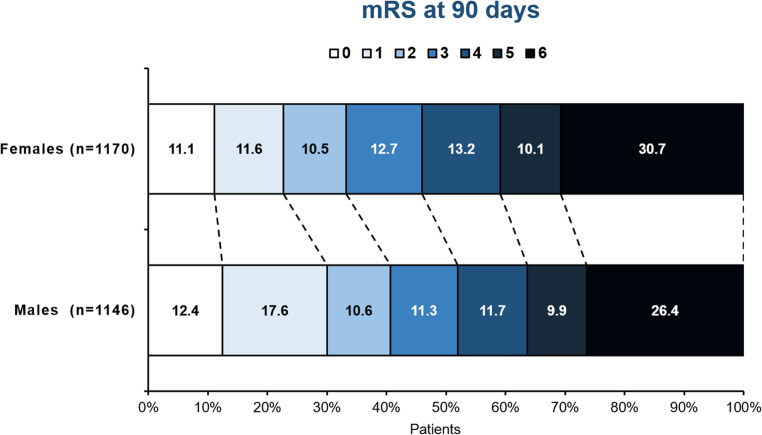
Distribution of the mRS scores by sex at 90 days. The distribution of the mRS scores of women and men 90 days after stroke thrombectomy is displayed. *mRS* modified Rankin Scale

Functional outcome 90 days after stroke thrombectomy differed significantly between women and men, with 32.2% females vs. 40.6% males (*p* < 0.001) being independent 90 days after stroke. Accordingly, mortality was higher in women (30.7% vs. 26.4%, *p* = 0.024) and a larger proportion of women had a poor outcome defined by a mRS 5 and 6 (40.8% vs. 36.4%, *p* = 0.033). Fig. 1 illustrates the distribution of each mRS score among females and males at 90 days.

### Binary Logistic Regression Analysis

**Fig. 2:**
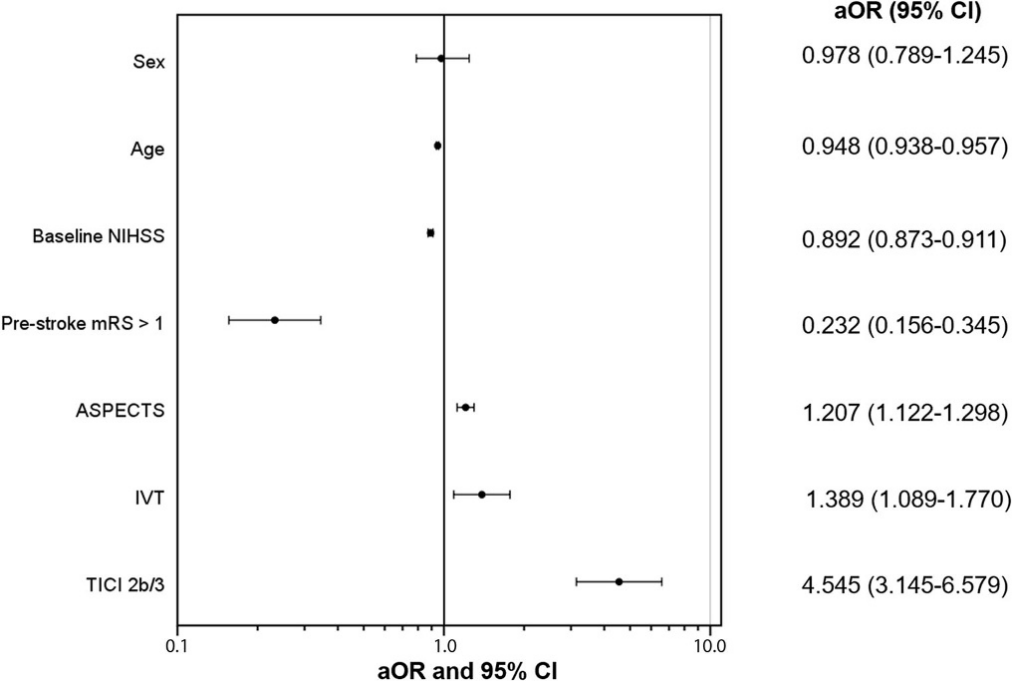
Predictors of outcome after stroke thrombectomy in clinical practice. Forest plot showing adjusted analyses (adjusted OR and 95% CI) of predictors of outcome after stroke thrombectomy in this patient cohort of clinical practice. Age, NIHSS, pre-stroke mRS, IVT, ASPECTS and successful reperfusion were significant predictors of independent outcome, while sex was not associated with outcome. *aOR* adjusted odds ratio, *CI* confidence interval, *NIHSS* National Institutes of Health Stroke Scale, *mRS* modified Rankin Scale, *IVT* intravenous thrombolysis, *ASPECTS* Alberta Stroke Program Early CT Score, *mTICI* modified thrombolysis in cerebral infarction score

In univariate logistic regression analyses, all variables of interest including sex showed a significant association with an independent outcome and death 90 days after stroke (Table 2a and Supplemental Table e2a). In model 1 of the multivariable regression analyses, a lower age, a lower baseline NIHSS score, successful reperfusion, prestroke functional independence and a concomitant IVT were significantly associated with independent outcome (Table 2b). In model 2 the ASPECTS predicted independent outcome in addition to the aforementioned variables (Table 2b, Fig. 2). Sex did not predict outcome in adjusted analyses.

**Table 2:** Predictors of independent outcome

**a) Univariate binary logistic regression analyses**		
	OR^a^ (95% CI)	*P* values
Sex (*n* = 2316)	0.729 (0.616–0.864)	*<0.001*
Age (*n* = 2316)	0.947 (0.940–0.954)	*<0.001*
Baseline NIHSS score (*n* = 2288)	0.898 (0.885–0.911)	*<0.001*
Prestroke mRS >1 (*n* = 2229)	0.152 (0.111–0.204)	*<0.001*
IVT (*n* = 2295)	1.582 (1.330–1.880)	*<0.001*
TICI2b/3 (*n* = 2268)	3.968 (2.967–5.291)	*<0.001*
ASPECTS (*n* = 1764)	1.244 (1.171–1.322)	*<0.001*
**b) Multivariable binary logistic regression analyses**		
		*P*
*Model 1: All independent variables except ASPECTS (n* *=* *2151)*		
Sex	0.986 (0.799–0.217)	0.897
Age	0.952 (0.944–0.961)	*<0.001*
Baseline NIHSS score	0.900 (0.855–0.915)	*<0.001*
Prestroke mRS >1	0.225 (0.159–0.319)	*<0.001*
IVT	1.645 (1.333–2.028)	*<0.001*
TICI2b/3	4.831 (3.460–6.757)	*<0.001*
*Model 2: All independent variables including ASPECTS (n* *=* *1667)*		
Sex	0.978 (0.789–1.245)	0.862
Age	0.948 (0.938–0.957)	*<0.001*
Baseline NIHSS	0.892 (0.873–0.911)	*<0.001*
Prestroke mRS >1	0.232 (0.156–0.345)	*<0.001*
IVT	1.389 (1.089–1.770)	*0.015*
ASPECTS score	1.207 (1.122–1.298)	*<0.001*
TICI2b/3	4.545 (3.145–6.579)	*<0.001*

*OR* odds ratio, *CI* confidence interval, *NIHSS* National Institutes of Health Stroke Scale, *mRS* modified Rankin Scale, *IVT* intravenous thrombolysis, *mTICI* modified thrombolysis in cerebral infarction score, *ASPECTS* Alberta Stroke Program Early CT Score

^a^OR >1 indicate higher probabilities of independent outcome (mRS0-2) at 90 days

The continuous variable symptom onset-to-recanalization was not associated with independent outcome when included in a secondary univariate and multivariate analysis (data not shown).

In line with this, a higher age, a higher baseline NIHSS score, a prestroke mRS score >1, no concomitant IVT and unsuccessful reperfusion were significant predictors of death (model 1; Supplemental Table e2b). After including the ASPECT score to this adjusted analysis, a lower ASPECTS was associated with death in addition to the abovementioned variables (model 2; Supplemental Table e2b). Sex did not predict outcome in this multivariable regression analysis. In a secondary analysis, symptom onset-to-recanalization was not associated with death (data not shown).

### Ordinal Regression Analyses

In univariate analyses, all variables of interest were significantly associated with the ordinal mRS score 90 days after stroke (Supplemental Table e3a). In adjusted analyses, a higher age, a higher baseline NIHSS score, a prestroke mRS score >1, no concomitant IVT and unsuccessful reperfusion were associated with worse outcome assessed by higher mRS scores at 90 days (model 1; Supplemental Table e3b). In model 2 a higher ASPECTS was associated with lower mRS scores (Supplemental Table e3b). In a secondary analysis, the variable symptom onset-to-recanalization did not show any association with the ordinal mRS score at 90 days (data not shown).

## Discussion

In this large prospective multicenter study of stroke patients treated by thrombectomy in clinical practice, we observed a worse functional outcome and higher mortality at 90 days in women; however, after adjustment for crucial confounders there was no association of sex with independent outcome.

These study results are consistent with previous studies, which did not find sex differences in outcome 90 days after stroke thrombectomy [6, 7]. In the HERMES pooled analysis of RCTs of stroke thrombectomy both women and men experienced comparable functional outcome and treatment effect of EVT was similar[6], despite women being older, similar as in our analysis. In contrast, in the MR CLEAN trial lower rates of functional independence and higher mortality were observed in women, together with a significant sex by treatment interaction with no significant treatment benefit in women [8]; however, these findings are solitary and have to be interpreted with caution, as the analysis was a post hoc subgroup analysis and the authors themselves suggested that the results may be explained by a play of chance.

Our analysis adds to the available studies of sex differences in outcome after stroke thrombectomy, as it is based on an independent large prospective registry of EVT in clinical practice. Trial populations are different from real-world patient cohorts resulting from the strict inclusion and exclusion criteria of clinical trials which are meant to ensure enrolment of those patients that are most likely to benefit from the investigational treatment. With respect to sex differences, women are frequently underrepresented in clinical trials [13], while in the real world stroke affects women and men in equal proportions. Moreover, stroke patients in trials are usually younger and have fewer comorbidities and less prestroke functional impairments than patients treated in clinical practice [1, 14, 15]. Comparing baseline characteristics of our study population to that from the HERMES analysis, some of these observations are confirmed. In HERMES, women were slightly underrepresented (47%), although in high-income countries women experience anterior circulation strokes more often [16]. In contrast, in our cohort 50.5% of patients were female and a higher proportion of women suffered from ischemic stroke of the anterior circulation. The analysis of the HERMES trial also showed similar rates of atrial fibrillation between women and men [6], while the prevalence of atrial fibrillation is usually higher in women [17], as it was in our study cohort. Moreover, the NIHSS score on admission was lower in women compared to men in the HERMES analysis, whereas the baseline NIHSS score did not differ by sex in our patient cohort. Of note, the MR CLEAN trial had less strict inclusion criteria than other RCTs of stroke thrombectomy, resulting in a patient cohort more comparable to real-world populations than other trial cohorts. Presumably, this might explain the similar outcome results with respect to the worse outcome in unadjusted analysis in MR CLEAN as compared to our cohort.

Similar to our analysis, a recent single center study of 279 patients in a real-world setting reported that females were less likely to be independent at 90 days after stroke thrombectomy than males [18]. In contrast, another single center study of stroke thrombectomy in clinical practice comprising 145 patients, found no sex differences in clinical outcome after EVT in acute ischemic stroke patients [19]. Small patient numbers as well as differences in baseline characteristics may account for the variability in the reported outcome results. In both studies mentioned above, women were older than men; however, the latter study included females with an equal prestroke disability compared to men, in contrast to our multicenter patient cohort in which prestroke disability differed significantly by sex with a larger proportion of women exhibiting functional disability before stroke. Notably, these parameters, i.e. age and prestroke functional impairment, are known predictors of outcome after EVT [20, 21].

After adjustment for confounders of interest (in both binary logistic and ordinal regression analyses), there was no significant association of sex and functional outcome in our study. Age and prestroke mRS were also identified as predictors of functional outcome in our patient cohort and showed significant differences between women and men. Thus, both variables may at least partially explain the observed sex differences in outcome. Similar findings were reported by the INSTRUCT collaboration [22].

The reason for the differences in age and prestroke functional status between women and men remains elusive. One interpretation is that women take care of more preventive efforts and have healthier lifestyles resulting in a longer stroke-free life. Thus, women are older when they suffer from first-ever stroke [22]. In consequence, more age-related diseases may occur accompanied by a higher prestroke disability [23] resulting in a less capacity to recover from stroke compared to men [22]. Moreover, the facts that a worse prestroke functional status in women may lead to less clear symptoms at onset time and that women usually take stroke symptoms less seriously, together may cause a late arrival to hospital and result in longer workflow times.

Notwithstanding, it is notable that other pivotal factors, such as the baseline NIHSS score, ASPECT score, the rates of prior IVT and successful reperfusion, in fact, did predict outcome in this patient cohort but showed no sex differences. Furthermore, in-hospital variables, such as the NIHSS and mRS score 24 h after intervention and at discharge as well as procedure times, the rate of adverse events and the rate of any intracerebral hemorrhage, were comparable between women and men. Thus, we cannot exclude that other variables which we did not collect might also add to sex differences in stroke outcome.

## Limitations

Our study has limitations. It lacks a patient group that received best medical treatment instead of EVT and, thus, we are not able to evaluate the effectiveness of EVT between women and men in clinical practice. Moreover, another important limitation is that some of the variables collected in the study had many missing values, in particular, the workflow times symptom onset-to-groin puncture and symptom onset-to-recanalization. However, this was considered in the statistical analysis. In addition, we cannot exclude a patient selection bias, since EVT was most likely performed in patients in whom a treatment benefit was assumed. Furthermore, we do not have data on other important, e.g. prestroke or postdischarge variables, which might have an impact on outcome in females.

## Conclusion

In a prospective multicenter registry study of endovascular stroke treatment in clinical practice, we observed a worse functional outcome in women, which appears to be explained by differences in baseline characteristics, such as age and prestroke modified Rankin scale, between women and men. As a consequence, sex was not an independent predictor of functional independence at 90 days after adjustment for confounders.

## Supplementary Information

Supplemental Tables containing group analyses between patients with and without outcome data as well as regression analyses about predictors of worse outcome and death 90 days after stroke
