# Supplementary material for: Sex Differences in Outcome After Thrombectomy for Acute Ischemic Stroke are Explained by Confounding Factors
Source: Clin Neuroradiol. 2020 Dec 21;31(4):1101–9. doi: 10.1007/s00062-020-00983-2 (PMC8648700; doi:10.1007/s00062-020-00983-2)
Supplement: Supplementary file 1 — Supplemental Tables containing group analyses between patients with and without outcome data as well as regression analyses about predictors of worse outcome and death 90 days after stroke [file 62_2020_983_MOESM1_ESM.docx]

**Electronic Supplemental Material – Tables**

**Table e1: Between-group analyses: patients with and without outcome data**

|  | **Patient subgroup with available outcome data (n=2316)** | **Patient subgroup without available outcome data (n=321)** | **P value** |
| --- | --- | --- | --- |
| **Age- median (IQR)** | 75 (64-82) | 75 (64-81) | 0.940 |
| **Baseline NIHSS – median (IQR)** | 15 (10-19) (n=2288) | 15 (10-19)  (n=313) | 0.174 |
| **Pre-stroke mRS > 1 – n (%)** | 436/2229 (19.6) | 46/293 (15.7) | 0.114 |
| **Living status before admission** |  |  | 0.709 |
| **Home – n (%)** | 1941/2195 (88.4) | 260/298 (87.2) |  |
| **Nursing at home – n (%)** | 92/2195 (4.2) | 12/298 (4) |  |
| **Nursing home – n (%)** | 162/2195 (7.4) | 25/298 (8.7) |  |
| **Pre-existing comorbidities** |  |  |  |
| **Arterial hypertension – n (%)** | 1732/2296 (75.4) | 244/316 (77.2) | 0.490 |
| **Hypercholesterinaemia – n (%)** | 774/2291 (33.8) | 107/311 (34.4) | 0.828 |
| **Diabetes Mellitus – n (%)** | 489/2299 (21.3) | 61/313 (19.5) | 0.468 |
| **Atrial fibrillation – n (%)** | 933/2293 (40.7) | 134/313 (42.8) | 0.474 |
| **Drip ‘n ship – n (%)** | 1103/2316 (47.6) | 161/321 (50.2) | 0.395 |
| **Anterior circulation infarction – n (%)** | 1994/2259 (88.6) | 268/306 (87.6) | 0.750 |
| **ASPECTS – median (IQR)** | 9 (7-10)  (n=1764) | 9 (7-10)  (n=252) | 0.313 |
| **IVT – n (%)** | 1286/2295 (56.0) | 171/315 (54.3) | 0.558 |

*Abbreviations: NIHSS, National Institutes of Health Stroke Scale; mRS, modified Rankin Scale; ASPECTS, Alberta Stroke Program Early CT Score; IVT, intravenous thrombolysis*

**Table e2: Predictors of death (mRS 6)**

1. **Univariate binary logistic regression analyses**

|  | OR^a^ (95% CI) | P values |
| --- | --- | --- |
| **Female sex (n= 2316)** | 1.232 (1.028-1.475) | *0.024* |
| **Age (n=2316)** | 1.058 (1.048-1.067) | *<0.001* |
| **Baseline NIHSS score (n=2288)** | 1.072 (1.058-1.086) | *<0.001* |
| **Pre-stroke mRS >1 (n=2229)** | 3.717 (2.990-4.630) | *<0.001* |
| **IVT (n=2295)** | 0.625 (0.521-0.750) | *<0.001* |
| **TICI2b/3 (n=2268)** | 0.331 (0.264-0.414) | *<0.001* |
| **ASPECTS (n=1764)** | 0.837 (0.792-0.886) | *<0.001* |

1. **Multivariable binary logistic regression analyses**

|  | OR^a^ (95% CI) | P values |
| --- | --- | --- |
| ***Model 1: All independent variables except ASPECTS (n=2151)*** | | |
| **Female sex** | 0.899 (0.722-1.120) | 0.343 |
| **Age** | 1.052 (1.041-1.063) | *<0.001* |
| **Baseline NIHSS score** | 1.072 (1.056-1.087) | *<0.001* |
| **Pre-stroke mRS >1** | 2.538 (1.984-3.257) | *<0.001* |
| **IVT** | 0.614 (0.496-0.761) | *<0.001* |
| **TICI2b/3** | 0.294 (0.227-0.381) | *<0.001* |
| ***Model 2: All independent variables including ASPECTS (n=1667)*** | | |
| **Female sex** | 0.827 (0.643-1.065) | 0.141 |
| **Age** | 1.057 (1.045-1.070) | *<0.001* |
| **Baseline NIHSS** | 1.093 (1.071-1.116) | *<0.001* |
| **Pre-stroke mRS >1** | 2.445 (1.835-3.257) | *<0.001* |
| **IVT** | 0.738 (0.577-0.943) | *0.015* |
| **ASPECTS score** | 0.860 (0.804-0.920) | *<0.001* |
| **TICI2b/3** | 0.347 (0.258-0.466) | *<0.001* |

^a^OR>1 indicate higher probabilities of independent outcome (mRS0-2) at 90 days
*Abbreviations: OR, odds ratio; CI, confidence interval; NIHSS, National Institutes of Health Stroke Scale; mRS, modified Rankin Scale; IVT, intravenous thrombolysis; mTICI, modified Thrombolysis in Cerebral Infarction Score; ASPECTS, Alberta Stroke Program Early CT Score*

**Table e3: Predictors of worse outcome assessed by higher mRS scores 90 days after stroke**

1. **Univariate ordinal regression analyses**

|  | OR^a^ (95% CI) | P values |
| --- | --- | --- |
| **Female sex (n= 2316)** | 1.279 (1.107-1.477) | *0.001* |
| **Age (n=2316)** | 1.051 (1.045-1.057) | *<0.001* |
| **Baseline NIHSS score (n=2288)** | 1.095 (1.083-1.107) | *<0.001* |
| **Pre-stroke mRS >1 (n=2229)** | 4.098 (3.356-5.000) | *<0.001* |
| **IVT (n=2295)** | 0.627 (1.330-1.880) | *<0.001* |
| **TICI2b/3 (n=2268)** | 0.299 (0.244-0.367) | *<0.001* |
| **ASPECTS (n=1764)** | 0.809 (0.771-0.849) | *<0.001* |

1. **Multivariable ordinal regression analyses**

|  | OR^a^ (95% CI) | P values |
| --- | --- | --- |
| ***Model 1: All independent variables except ASPECTS (n=2151)*** | | |
| **Female sex** | 0.968 (0.799-0.828) | 0.695 |
| **Age** | 1.044 (1.038-1.051) | *<0.001* |
| **Baseline NIHSS score** | 1.094 (1.082-1.107) | *<0.001* |
| **Pre-stroke mRS >1** | 2.865 (2.315-3.546) | *<0.001* |
| **IVT** | 0.615 (0.526-0.719) | *<0.001* |
| **TICI2b/3** | 0.252 (0.203-0.314) | *<0.001* |
| ***Model 2: All independent variables including ASPECTS (n=1667)*** | | |
| **Female sex** | 0.996 (0.839-1.201) | 0.966 |
| **Age** | 1.049 (1.042-1.058) | *<0.001* |
| **Baseline NIHSS** | 1.108 (1.092-1.125) | *<0.001* |
| **Pre-stroke mRS >1** | 2.755 (2.160-3.509) | *<0.001* |
| **IVT** | 0.725 (0.606-0.867) | *<0.001* |
| **ASPECTS score** | 0.832 (0.790-0.877) | *<0.001* |
| **TICI2b/3** | 0.273 (0.214-0.349) | *<0.001* |

^a^OR>1 indicate higher probabilities of independent outcome (mRS0-2) at 90 days
*Abbreviations: OR, odds ratio; CI, confidence interval; NIHSS, National Institutes of Health Stroke Scale; mRS, modified Rankin Scale; IVT, intravenous thrombolysis; mTICI, modified Thrombolysis in Cerebral Infarction Score; ASPECTS, Alberta Stroke Program Early CT Score*
